# Simulating the landscape eco-evolution of host-pathogen systems with CDMetaPOP

**DOI:** 10.1016/j.ecoinf.2026.103892

**Published:** 2026-08

**Authors:** Erin L. Landguth, Allison Williams, Marissa Roseman, Miracle Amadi, Baylor Fain, Marcel Kouete, Rhys A. Farrer, Amy J. Haeseler, Orly Razgour, Chris Richardson, Byron Weckworth, Julie Weckworth, Flora Whiting-Fawcett, Duncan Wilson, Casey C. Day

**Affiliations:** a Center for Population Health Research, School of Public and Community Health Sciences, University of Montana, Missoula, United States of America; b School of Environment & Natural Resources, College of Food, Agriculture, and Environmental Sciences, The Ohio State University, Columbus, United States of America; c UT School of Engineering Sciences, Lappeenranta-Lahti University of Technology (LUT), Lappeenranta, Finland; d MRC Centre for Medical Mycology, Biosciences, University of Exeter, Exeter, UK; e Biosciences, University of Exeter, Exeter, UK; f Department of Biology, Boston University, United States of America

**Keywords:** Individual-based model, Infectious disease modeling, Landscape connectivity, Landscape genetics, Eco-evolutionary dynamics, Simulation software, Host-pathogen interactions

## Abstract

Simulating the eco-evolutionary feedbacks between wildlife hosts and pathogens in complex landscapes remains a major computational challenge. While universal simulation languages enable custom model development, few integrated frameworks exist that mechanistically link disease dynamics, landscape-scale demography, and forward-time population genetics. We address this gap by introducing CDMetaPOP-Disease, an open-source, spatially explicit, individual-based demo-genetic module. The module's primary informatics advance is its native ability to mechanistically link individual genotypes to epidemiological transition rates, enabling the simulation of host disease response strategies, such as resistance (reducing infection probability) and tolerance (reducing mortality). We verified the module's core features against classic SIR-type models, achieving high fidelity (Nash-Sutcliffe Efficiency ≥ 0.98) to theoretical expectations. We demonstrated the model's utility by simulating the emergence of a novel pathogen in a realistic bat-pathogen system, showcasing the tool's power to track both pathogen diffusion and the resulting spatial-genetic signatures of host adaptation. Optimized for performance and modularity, CDMetaPOP-Disease provides a flexible platform for the ecological informatics community to investigate complex feedbacks and inform proactive management of infectious diseases in realistic landscapes.

## Highlights

• Introduces CDMetaPOP-Disease, an integrated eco-evolutionary software module.
• Mechanistically links individual genotypes to fitness in complex landscapes.
• Validates stochastic outcomes against deterministic SIR-type models.
• Models the spatial evolution of host disease resistance and tolerance strategies.
• Open-source Python framework optimized for large-scale metapopulation simulations.

## 1 Introduction

Predicting the evolutionary trajectory of host-pathogen systems in realistic landscapes remains a critical computational and informatics challenge in ecology (Biek and Real, 2010; Kozakiewicz et al., 2018). While landscape genomics has advanced our ability to identify loci under selection (Aguirre-Liguori et al., 2021; Razgour et al., 2018), few frameworks possess the architecture required to simulate the forward-time demo-genetic processes (the interaction between demographic processes and genetic variation across space) of host adaptation to disease. An ecological informatics gap remains: the lack of modular software tools that natively integrate these high-dimensional, eco-evolutionary feedbacks. These feedbacks are often driven by selection on specific defense traits: primary resistance (the ability of a host to reduce the probability of infection) and tolerance (the ability of a host to reduce fitness costs, such as disease-induced mortality, once an infection has occurred) (Råberg et al., 2007; Roy and Kirchner, 2000).

Existing computational approaches for modeling these dynamics generally fall into two categories: simulation platforms and specialized software packages. Platforms like SLiM4 (Messer 2013; Haller & Messer 2019) or NetLogo (Wilensky 1999) provide powerful, general-purpose environments for custom model development. However, implementing a spatially explicit, demo-genetic epidemic model in these environments requires significant user-end programming to mechanistically link landscape resistance, individual-level demography, and epidemiological state transitions. For many researchers, this represents a significant technical barrier, as the informatics burden of managing state-transition logic alongside spatial-genetic data can be prohibitive. Conversely, specialized R packages for mathematical epidemiology (e.g., epinet, EpiModel) are highly effective for the modeling of transmission but lack the ability to handle high-resolution landscape resistance or forward-time simulation of multi-locus variation. Thus, an integrated informatics solutions that provide a mechanistic link between host genetics, spatial ecology, and epidemiology in a single, ready-to-use framework is needed.

To address this gap, we developed a flexible disease module for the CDMetaPOP modeling platform, a spatially explicit, individual-based, demo-genetic simulator (Day et al., 2023; Landguth et al., 2016). CDMetaPOP is designed with an object-oriented architecture that facilitates the management of thousands of individual agents, each carrying unique genetic and epidemiological attributes. The module's primary advancement is the incorporation of a compartmental modeling framework into a mechanistic, spatially explicit, individual-based, open-population simulator, to support modeling the evolution of host disease response strategies across time and space without the need for bespoke code development. By optimizing the data structures used to track state transitions and genetic inheritance, this ready-to-use integrated framework lowers the technical barrier to performing complex multi-scale simulations of host-pathogen systems while maintaining the computational efficiency required for range-wide analyses.

In this paper, we first verify the module's core epidemiological logic against classical deterministic models, which demonstrates its high fidelity to theoretical expectations. We then showcase its eco-evolutionary capabilities by simulating how selection for disease resistance and tolerance impacts epidemiological outcomes and drives the fixation of adaptive alleles. Finally, we illustrate its practical utility by simulating the emergence of a novel pathogen in a range-wide system for the cave myotis (*Myotis velifer*), showcasing the tool's power to model both disease spread and the resulting spatial-genetic signature of host adaptation in realistic landscapes. Through these demonstrations, we highlight how integrated informatics tools can empower researchers to test hypotheses that were previously computationally inaccessible.

## 2 Methods and results

CDMetaPOP is a spatially explicit, individual-based, demo-genetic simulator (Day et al., 2023; Landguth et al., 2016). The base platform blends demographic processes (e.g., growth, reproduction, mortality) with landscape-governed movement (e.g., isolation-by-resistance) while tracking individual-level genotypes. Box 1 provides a concise recap of the core spatial, evolutionary, and demographic capabilities of the CDMetaPOP platform.

Box 1 **Core capabilities of CDMetaPOP.**

CDMetaPOP (Landguth et al., 2017) is a multi-species (Day et al., 2023), individual-based, spatially explicit, landscape demogenetic forward-time simulator designed to model metapopulation dynamics across land- (e.g., Forester et al., 2023) and aquatic-scapes (e.g., Escalante et al., 2018). Core Features include:

**Demography:** Simulates individual growth, age/stage/size-based structure, and complex vital rates (maturation, fecundity, and diverse mortality options including density-dependence) (e.g., Day et al., 2018; Kristensen et al., 2018).

**Genetics:** Tracks neutral and adaptive multilocus genotypes (e.g., Landguth et al., 2020) via mendelian inheritance, mutation, local adaptation via a novel allele or pre-existing allele, plasticity (e.g., Seaborn et al., 2023), hybridization, and introgression between species (e.g., Menon et al., 2020; Sullivan et al., 2026).

**Spatial Dynamics:** Movement (dispersal, migration, and straying) is governed by high-resolution resistance-to-movement surfaces (e.g., Bernos et al., 2023; Day et al., 2024). Supports asymmetrical resistance (e.g., wind; Landguth et al., 2017 or river current; Mims et al., 2019).

**Temporal Variability:** Parameters and processes can vary through time, enabling the simulation of dynamic landscapes, such as changing climate or land-use conditions (e.g., Araya-Donoso et al., 2022; Nathan et al., 2017).

**Disease Module (New):** Integrates flexible compartmental state transitions (e.g., SIR, SEIR) and mechanistically links genotypes to defense traits like resistance and tolerance (this study).

### 2.1 The CDMetaPOP-disease module

To ensure reproducibility and accessibility, full documentation, example input files, and parameters are available at the GitHub repository.

Utilizing CDMetaPOP's modular design, the disease module provides an integrated compartmental framework based on classic SIR models (Kermack & McKendrick 1927). The module operates on an update cycle. In each time step, individuals undergo demographic processes (e.g., mortality, movement, and reproduction) followed by epidemiological state transitions. These transitions are modeled as Bernoulli trials for each individual, where the probability of transition (e.g., S to I) is governed by user-defined rates and the initial conditions (Table 1, Table 2). In the case of transmission, the module supports frequency-dependent transmission by default (governed by the local proportion of infectious agents), with flexibility for users to implement density-dependent effects by scaling transition rates. The module includes four primary features designed for wildlife epidemiology:

**Table 1:** Definition of state variables and parameter values as transition rates 1 / time.

States	Description
N	Total population
S	Number of Susceptible individuals
E	Number of Exposed individuals
I	Number of Infected individuals - those that are spreading infection
R	Number of Recovered individuals
D	Number of Dead individuals
P	Amount of Pathogen or contaminant in the environment
Parameters	Description
α	Rate of pathogen or contaminant shedding into the environment
ꞵ	Transmission rate where subscript *d* = direct and subscript *i* = indirect
k	Death rate
γ	Recovery rate
ε	Incubation rate
φ	*Re*-susceptibility rate
μ	Pathogen or contaminant decay rate out of the environment

**Table 2:** Validation, selection and example scenarios. Parameters are transition rates 1 / time.

Scenario	Compartmental diagram, equations, initial conditions and parameter values
Validation SIR	Equations: dS/dt = − βSI/N; dI/dt = βSI/N − γI; dR/dt = γI; N = S + I + R Initial Conditions: N = 100; S0 = 90; I0 = 10; R0 = 0 Parameters: β = 0.5; γ = 0.2
Validation SIRS	Equations: dS/dt = − βSI/N + φR; dI/dt = βSI/N − γI; dR/dt = γI − φR; N = S + I + R Initial Conditions: *N* = 100; S0 = 90; I0 = 10; R0 = 0 Parameters: β = 0.5; γ = 0.2; φ = 0.2
Validation SEIR	Equations: dS/dt = − βSI/N; dE/dt = βSI/N − εE; dI/dt = εE − γI; dR/dt = γI; N = S + E + I + R Initial Conditions: N = 100; S0 = 90; E0 = 0; I0 = 10; R0 = 0 Parameters: β = 0.5; ε = 0.5; γ = 0.2
Validation SIRP	Equations: dS/dt = − βdSI/N − βiSP; dI/dt = βdSI/N + βiSP − γI; dR/dt = γI; dP/dt = αI − μP; N = S + I + R Initial Conditions: N = 100; S0 = 90; I0 = 10; R0 = 0; P0 = 10 Parameters: βd = 0.25; βi = 0.3; γ = 0.05; α = 0.1; μ = 0.015
Selection	Equations: dS/dt = − βSI/N; dI/dt = βSI/N − γI − κI; dR/dt = γI; dD/dt = κI Initial Conditions: *N* = 1000; S0 = 900; I0 = 10; R0 = 0; D0 = 0 Disease Neutral Parameters: β = 0.8; γ = 0.1; κ = 0.1 Disease Resistant Parameters: β = 0.01; γ = 0.1; κ = 0.1 Disease Tolerant Parameters: β = 0.8; γ = 0.1; κ = 0 Disease Resistant & Tolerant Parameters: β = 0.01; γ = 0.1; κ = 0
Example Applications	Initial Conditions: See Appendix for patch specific values Disease Neutral Parameters: βd=0.2; βi=0.9; φ=0.01; κ =0.1; μ=0; α=0.5;α=0.5 Disease Resistant parameters: βd = 0; βi = 0; φ = 0.01; κ = 0.1; μ = 0; α = 0.5; α=0.5 Disease Tolerant Parameters: βd = 0.2; βi = 0.9; φ = 0.01; κ = 0; μ = 0; α = 0.5; α=0.5 Disease Resistant + Tolerant Parameters: βd = 0; βi = 0; φ = 0.01; κ = 0; μ = 0; α= 0.5; α=0.5

Flexible State Definitions: Users can define any number of compartments (e.g., S, E, I, R, V, D) and/or load classes (I_1_, I_2_, I_3_, …, I_n_) and the transition matrix between them.

Transmission Modes: Supports vertical transmission, direct host-to-host transmission, and indirect transmission via an environmental reservoir (P), including pathogen shedding and decay rates.

Life-History Integration: Accommodates diploid organisms with overlapping or non-overlapping generations and sexual or asexual reproduction.

Native Data Output Structure: From an informatics perspective, the software generates individual-level attribute files where a dedicated state column tracks the disease compartment of every agent, alongside a dedicated summary file (summary_popAllTime_DiseaseStates.csv) that provides time-series snapshots of the epidemic's progression at multiple life-cycle stages.

### 2.2 Simulating Eco-evolution: genotype-linked disease response

The module's primary eco-evolutionary innovation leverages CDMetaPOP's existing infrastructure for natural selection (Landguth et al., 2012, Landguth et al., 2020) to model the evolution of host disease response. The platform can mimic the introduction of a “novel allele” (e.g., mutation) or “pre-existing allele” (e.g., standing genetic variation) simulation (e.g., Creech et al., 2017). Users can mechanistically link an individual's diploid genotype at one or more loci to any probabilistic transition rate in the disease model (see 2.5, 2.6 for examples). This allows for explicit simulation of spatial selection on disease response strategies, such as resistance (e.g., linking a homozygous ‘RR’ genotype to a reduced S-to-I transition rate) or tolerance (e.g., linking a homozygous ‘TT’ genotype to a reduced disease-induced mortality rate). This flexibility enables users to simulate local adaptation and test hypotheses about the evolution of diverse genetic defense mechanisms without requiring the development of custom inheritance or selection algorithms. Furthermore, by mapping genotypes across multiple parameters simultaneously, users can explicitly model pleiotropic trade-offs or “cost functions” (e.g., Boots et al., 2009; Duffy et al., 2008). For instance, a researcher can simulate a scenario where a resistance-conferring allele at one locus also imposes a fitness cost, such as a reduction in fecundity or slower individual growth, enabling a mechanistic investigation into the long-term maintenance of defense strategies within realistic landscapes.

### 2.3 Verification against theoretical expectations

**Fig. 1:**
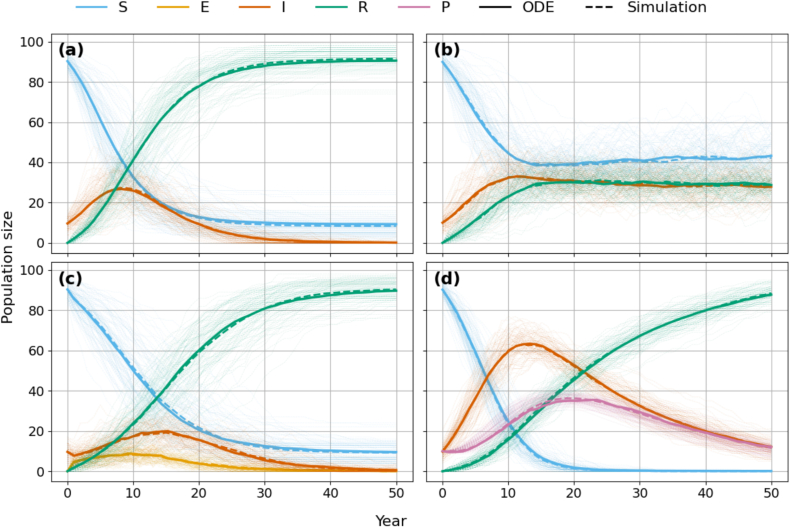
Theoretical and simulated expectations for (a) SIR, (b) SIRS, (c) SEIR, and (d) SIRP. Shown are the mean of 100 replicates for the mean of the CDMetaPOP simulations (bold dashed line), each individual CDMetaPOP replicate (light lines), and the ODE SIR-type curves (solid lines), over 50 time units. For the different SIR-type models shown: Susceptible (S, blue lines), Exposed (E, orange lines), Infected (I, red lines), Recovered (R, green lines), and Pathogen (P, purple lines). See Table 3, Table 4 for parameters and initial conditions used. (For interpretation of the references to colour in this figure legend, the reader is referred to the web version of this article.)

To verify the module's core epidemiological function against established theory, we tested its ability to replicate the dynamics of four common Ordinary Differential Equation (ODE) models: SIR, SIRS, SEIR, and SIRP (environmental pathogen reservoir). We configured CDMetaPOP-Disease to mirror the assumptions of these classical models (e.g., closed population size, no spatial processes), initial conditions and parameters (see Table 1, Table 2). The mean of 100 stochastic replicates from our individual-based module accurately matched the output of the theoretical models solved via the tau-leap method (Fig. 1). For each compartment of each model the following comparison statistics were calculated using the python package *permetrics*: Normalized Root Mean Square Error (NRMSE), Nash Sutcliffe Efficiency (NSE), and Kling-Gupta Efficiency (KGE) (Table 3). While these verification scenarios assume a closed population, the subsequent “Use Case” (2.5, 2.6) demonstrates the module's full integration with complex demo-genetic processes, including overlapping generations, movement, and age-specific mortality.

**Table 3:** Comparison statistics for each compartment of each model in Section 2.3. For NRMSE, smaller values indicate higher accuracy, with 0 being a perfect match. Conversely, NSE and KGE values closer to 1 indicate higher accuracy; thresholds of NSE = 0 and KGE = −0.41 serve as thresholds where model predictions are no more informative than the mean of the observed data.

Model	Compartment	Normalized Root Mean Square Error (NRMSE)	Nash Sutcliffe Efficiency (NSE)	Kling Gupta Efficiency (KGE)
SIR	S	0.03	1.00	0.96
SIR	I	0.06	1.00	0.97
SIR	R	0.03	1.00	0.98
SIRS	S	0.08	0.99	0.97
SIRS	I	0.15	0.98	0.96
SIRS	R	0.09	0.99	0.98
SEIR	S	0.05	1.00	0.98
SEIR	E	0.10	1.00	0.96
SEIR	I	0.12	0.99	0.96
SEIR	R	0.04	1.00	0.97
SIRP	S	0.01	1.00	0.97
SIRP	I	0.04	1.00	0.97
SIRP	R	0.02	1.00	0.99
SIRP	P	0.07	0.99	0.98

### 2.4 Parameter estimation via data fitting

To extend our verification, we assessed the framework's capability for inverse modeling by fitting CDMetaPOP to synthetic datasets to produce accurate parameter estimates. We used the stochastic output from the SIR and SIRS scenarios (Fig. 1a, b) as synthetic observational data. The best-fit was determined by minimizing the sum of squared residuals (SSR): SSR = $\sum_{i=1}^{n}{\left(y_{i}-{\hat{y}}_{i}\right)}^{2}$, where $y_{i}$ represents a synthetic data point and ${\hat{y}}_{i}$ is a data point from the mean of the 100 CDMetaPOP simulations.

**Fig. 2:**
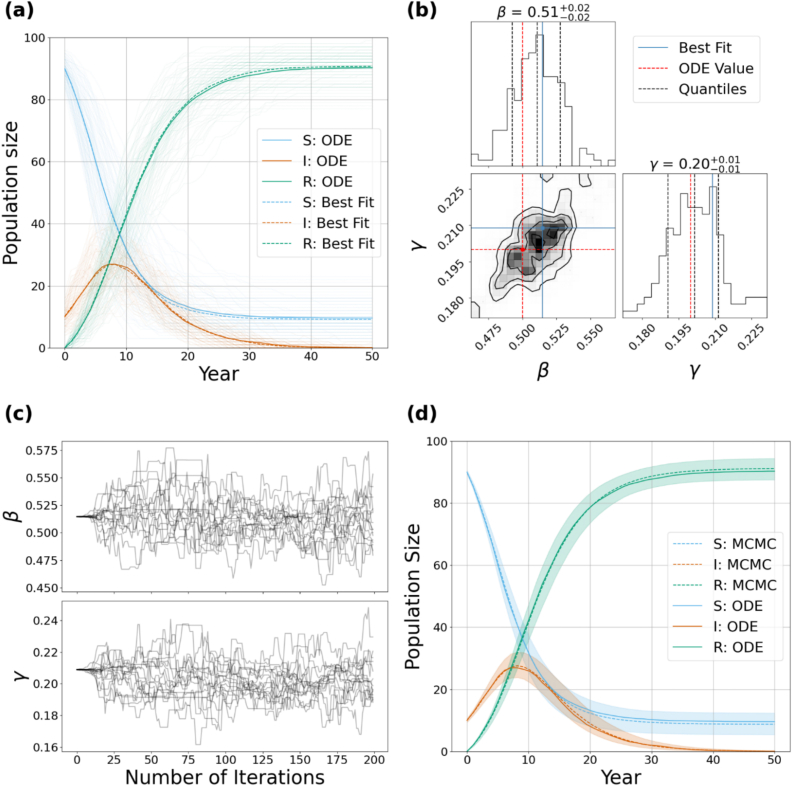
Parameter Fitting: (a) CDMetaPOP fit to synthetic data produced by an SIR ODE model, (b) corner plot from MCMC uncertainty estimation, (c) plot of “walkers” used in MCMC sampling to show parameter space exploration, and (d) CDMetaPOP with 95% confidence intervals compared to synthetic data: Shown in (a) is the best fit from minimizing the SSR between the mean of 100 CDMetaPOP simulations and synthetic data produce by a SIR ODE model. (b) shows the corner plot result of MCMC uncertainty estimation with 16 “walkers” and 201 iterations per walker, with the best fit parameters (β = 0.51, and γ = 0.21) as the initial guess, (c) shows the trace plot of each “walker” showing how the parameter space was explored, (d) shows the comparison between the SIR ODE model and the CDMetaPOP simulations with 95% confidence intervals generated from the MCMC sampling.

**Fig. 3:**
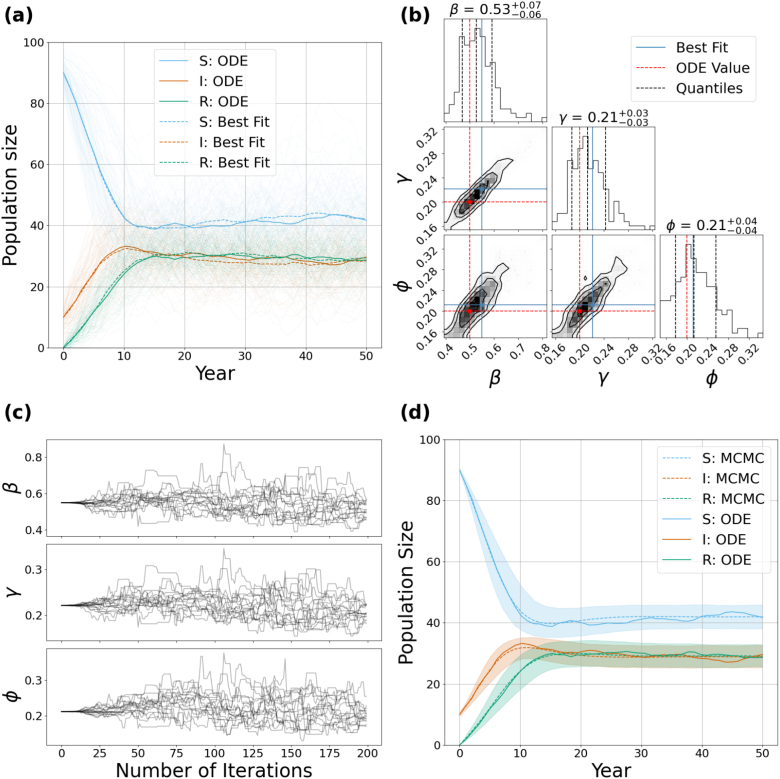
Parameter Fitting: (a) CDMetaPOP fit to synthetic data produce by an SIRS ODE model, (b) corner plot from MCMC uncertainty estimation, (c) plot of “walkers” used in MCMC sampling to show parameter space exploration, and (d) CDMetaPOP with 95% confidence intervals compared to synthetic data: Shown in (a) is the best fit from minimizing the SSR between the mean of 100 CDMetaPOP simulations and synthetic data produce by a SIRS ODE model. (b) shows the corner plot result of MCMC uncertainty estimation with 16 “walkers” and 201 iterations per walker, with the best fit parameters (β = 0.55, γ = 0.22, and ɸ = 0.21)as the initial guess, (c) shows the trace plot of each “walker” showing how the parameter space was explored, (d) shows the comparison between the SIRS ODE model and the CDMetaPOP simulations with 95% confidence intervals generated from the MCMC sampling.

To quantify parameter uncertainty, we implemented an ensemble-based Markov Chain Monte Carlo (MCMC) approach. The estimation pipeline was developed as a modular Python script utilizing the function *minimize* (package scipy) and the function *EnsembleSampler* (package *emcee*). During the fitting process, the transmission (ꞵ) and recovery (γ) parameters were allowed to vary for the SIR data, and the transmission (ꞵ), recovery (γ), and waning immunity (ɸ) parameters were allowed to vary for the SIRS data. During the uncertainty estimation, the MCMC sampler used uniform priors ($\cup \left(01\right)$) for each parameter, 16 “walkers” initialized uniformly around the best-fit starting point and ran for 200 iterations per walker (totaling 3216 simulations; 16*201). Median parameter values and credible intervals were calculated from the pooled posterior distributions, after discarding the first 100 iterations as burn-in and subsampling every 10th iteration to mediate autocorrelation. The resulting fits and estimated parameters (Fig. 2, Fig. 3**, and** Table 4) demonstrate that the framework can accurately recover true, underlying epidemiological parameters from stochastic data.

**Table 4:** The true parameters from the SIR and SIRS scenarios (Fig. 1a, b), the best fit parameters from (Figs. 2a, 3a), and the median parameters and uncertainties from (Fig. 2b, b).

Model	True Parameters	Best Fit	Median Parameters	Uncertainty
SIR	β = 0.5 γ = 0.2	β = 0.51 γ = 0.21	β = 0.51 γ = 0.20	β: + − 0.02 γ: + − 0.01
SIRS	β = 0.5 γ = 0.2 ɸ = 0.2	β = 0.55 γ = 0.22 ɸ = 0.21	β = 0.53 γ = 0.21 ɸ = 0.21	β: + − 0.06 γ: + − 0.03 ɸ: + − 0.04

### 2.5 Use case, demonstration of evolutionary dynamics

**Fig. 4:**
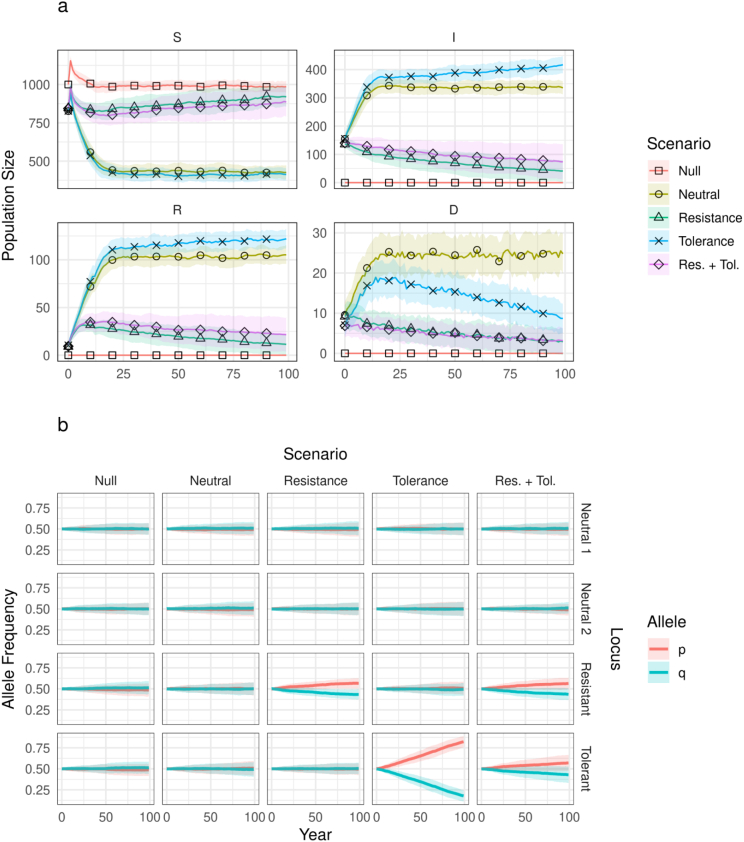
Selection scenarios: (a) population dynamics and (b) allele frequencies in a single aspatial population with overlapping generations: Five simulation scenarios testing selection-driven alleles response to disease dynamics over 100 years and averaged across 100 stochastic simulations. (a) Shows the mean (line) ± 1 SD (shaded area) number of individuals (y-axis) in the S (Susceptible), I (Infected), R (Recovered) and D (Dead) disease states over time. Five scenarios are differentiated by line colour and point symbols every 10 years. The Null scenario (square symbol red line) displays population dynamics without disease introduction. In all other scenarios, disease dynamics were occurring and individuals randomly inherited alleles associated with resistance, tolerance, or both. The initial increase is due to the initial population age structure stabilizing relative to the vital rates specified in the ClassVars file. (b) Displays the mean (line) ± 1 SD (shaded area) allele frequencies (left y axis) over time (bottom x axis) for the same five scenarios (top x axis) for each locus (right y axis), including two neutral loci, one resistant locus, and one tolerant locus. *p* - first allele and *q* - second allele. (For interpretation of the references to colour in this figure legend, the reader is referred to the web version of this article.)

To demonstrate CDMetaPOP's eco-evolutionary advancements, we simulated a SIRD model in a single population (*N* = 1000) over 100 overlapping generations, comparing a neutral scenario (disease present, no selection) to scenarios with selection on dominant alleles for resistance, tolerance, or both. As hypothesized, the adaptive alleles for resistance and tolerance showed a clear trend toward fixation, while neutral loci fluctuated randomly. The evolutionary response fundamentally altered disease outcomes: the resistance scenario effectively contained the disease, while the tolerance scenario mitigated mortality but allowed the pathogen to persist, resulting in a population of primarily tolerant, recovered individuals (Fig. 4).

### 2.6 Use case, application to a spatial eco-evolutionary system

**Fig. 5:**
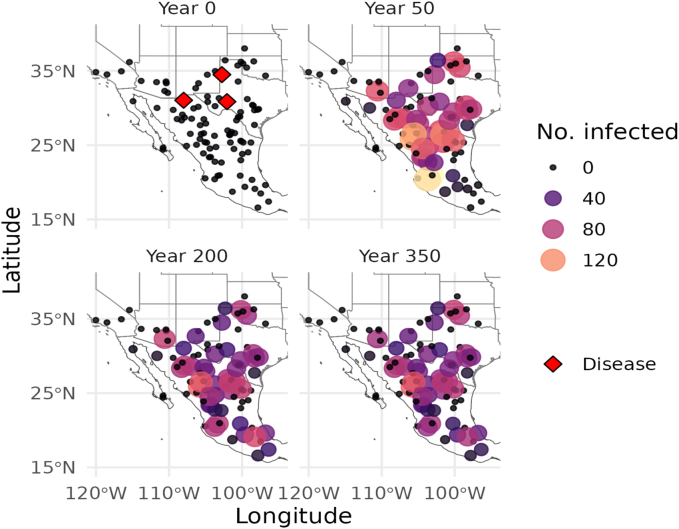
Example application, simulating disease spread on a range-wide *Myotis velifer* system model established in Roseman & Williams et al. 2025. Black circles in the top-left panel represent 100 patches, representing bat roosting locations. Three red diamonds display where the environmental pathogen begins in three hypothetical locations. Panels for years 50, 200, and 350 show the spread of infected individual bats at roosting locations as the simulation progresses. No adaptive alleles were introduced in this simulation. (For interpretation of the references to colour in this figure legend, the reader is referred to the web version of this article.)

To demonstrate the module's realism in a contextualized landscape, we simulated the emergence and eco-evolutionary consequences of a novel pathogen in a range-wide system for the Cave Myotis (*Myotis velifer*) (Roseman & Williams et al. in prep). The model includes spatial-demographic realism, with 100 patches (caves), age-specific vital rates, and seasonal movement. We first simulated the introduction of an environmental pathogen, showing its rapid diffusion through high-density patches and slower spread to isolated patches over 350 years (Fig. 5).

**Fig. 6:**
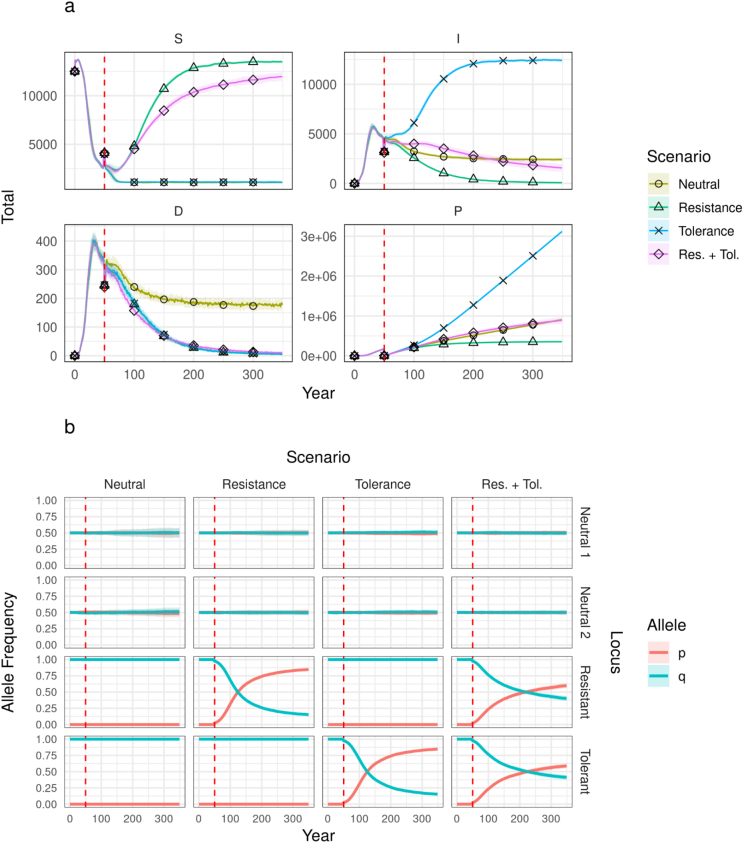
Example application: (a) population dynamics and (b) allele frequencies of the *Myotis velifer* metapopulation following disease introduction. Metapopulation responses to an environmental pathogen introduction and 350 total years of disease spread averaged across 25 stochastic simulations. At year 50 (red vertical lines), 150 individuals across three patches were added to the simulation carrying novel neutral or adaptive alleles. (a) Shows the mean (line) ± 1 SD (shaded area) number of individuals (y-axis) in the S (Susceptible), I (Infected), and D (Dead) disease states, as well as P, the total pathogen load across all patches over time (x-axis). Four simulation scenarios are differentiated by line colour and point symbols. The neutral scenario (yellow circles) represents the addition of individuals with neutral alleles; all others received adaptive alleles. (b) Displays the mean (line) ± 1 SD (shaded area) allele frequencies (left y axis) over time (bottom x axis) for the same four scenarios (columns) for each locus (rows), including two neutral loci, one resistant locus, and one tolerant locus. *p* - first allele and *q* - second allele. In scenarios where *p* = 0 and *q* = 1 throughout, the first allele (*p*) was never introduced and remained absent over time. (For interpretation of the references to colour in this figure legend, the reader is referred to the web version of this article.)

Next, we repeated the simulation but introduced adaptive alleles (for resistance, tolerance, or both) at the three introduction sites after 50 years. The results showed that selection, mediated by host movement, drove a spatial-genetic replacement of the ancestral allele with the adaptive variant (Fig. 6b). Interestingly, the rate of allele fixation was slower when both resistance and tolerance segregated simultaneously, suggesting that multiple defensive strategies can interact to dampen the selective pressure on individual loci. This evolution, in turn, reshaped the spatial disease dynamics, with resistance leading to a metapopulation-wide decline in infections and tolerance leading to a decline in mortality (Fig. 6).

## 3 Discussion

We have introduced and verified against established theory a flexible, open-source informatics module for CDMetaPOP that mechanistically links host genetics, spatial demography, and epidemiology. The primary advance of this framework is its ability to simulate the eco-evolutionary dynamics of host-pathogen systems, which allows researchers to investigate selection on traits like resistance and tolerance in realistic landscapes. By providing an integrated platform, CDMetaPOP-Disease moves beyond static snapshots, enabling forward-time hypothesis testing about how wildlife populations adapt to emerging infectious diseases in high-resolution spatial contexts.

### 3.1 An integrated framework for eco-epidemiology

There is a lack of “off-the-shelf” tools that can simultaneously handle complex landscapes, individual-level demography, and genomic variation. While platforms such as SLiM4 (Haller & Messer 2019) and NetLogo (Wilensky 1999) provide the flexibility to build such models, they require significant user-end programming and a deep understanding of simulation architecture to link these disparate processes. In contrast, CDMetaPOP-Disease offers a native, mechanism-based framework that eliminates the need for bespoke script development. This integration is particularly important when modeling the emergence of spatial-genetic signatures, where the interaction between host movement (connectivity) and pathogen spread can create complex selective surfaces that are difficult to capture in statistical or non-spatially explicit models, as well as lower-level programming environments.

### 3.2 Advancing beyond aspatial eco-evolutionary theory

While the fundamental trade-offs between resistance and tolerance are theoretically well-established (e.g., Roy and Kirchner, 2000), CDMetaPOP-Disease enables researchers to investigate these dynamics within the context of landscape complexity, a frontier that remains largely inaccessible to deterministic models. Our framework allows for the exploration of how landscape connectivity and corridor configurations shape the “selective surface” for defense traits. For example, users can simulate how the spatial diffusion of a pathogen across heterogeneous landscapes creates a mosaic of selective pressures, potentially leading to localized resistance in high-connectivity “hubs” while tolerance strategies persist in isolated “refugia.”

By integrating individual-level demography with forward-time genetics, the model captures stochastic eco-evolutionary feedbacks that deterministic systems omit. This includes investigating how selection for tolerance might inadvertently sustain higher environmental pathogen loads, thereby altering the selective landscape for neighboring subpopulations via source-sink dynamics. These spatially explicit insights provide a mechanistic basis for predicting population persistence in fragmented habitats, representing a significant move toward proactive, landscape-aware infectious disease management.

### 3.3 Applications and life-history flexibility

While demonstrated here with a bat-pathogen system, this module is generalizable across taxa and systems. The software's support for diploidy and overlapping generations makes it suitable for a wide range of vertebrates and plants, while its flexibility in state definition allows for the simulation of diverse management scenarios, such as the spatial deployment of vaccines or the impact of environmental contaminants. Beyond wildlife conservation, this tool has potential informatics applications in fisheries, agriculture, and livestock disease management, where understanding the spatial spread of resistance is paramount.

### 3.4 Limitations and future directions

Despite its utility, our framework has limitations that serve as avenues for future informatics development. For example, currently the module treats pathogen pressure as a static selective force. While host evolution is modeled in detail, the pathogen itself does not yet possess its own evolving genome within this framework. Implementing true host-pathogen co-evolution, where both species adapt in response to the other, remains a key future goal. Furthermore, while the current module focuses on discrete loci, future versions could incorporate automated pipelines to ingest high-throughput sequencing data (e.g., RAD-seq or whole-genome sequencing; WGS) into model parameterization. Future directions also include additional modes of transmission, such as sexual and vector borne.

Ultimately, as the ecological and human costs of wildlife diseases continue to escalate, mechanism-based forecasting tools that lower the barrier to complex modeling are essential. CDMetaPOP-Disease provides the ecological informatics community with a timely and flexible platform to investigate these complex feedbacks and inform proactive conservation and management strategies in a rapidly changing world.

## AI disclosure

AI assisted tools were used to check grammar in the manuscript.

## CRediT authorship contribution statement

**Erin L. Landguth:** Writing – review & editing, Writing – original draft, Validation, Supervision, Software, Resources, Project administration, Methodology, Investigation, Funding acquisition, Data curation, Conceptualization. **Allison Williams:** Writing – review & editing, Visualization, Investigation, Formal analysis, Data curation. **Marissa Roseman:** Writing – review & editing, Visualization, Investigation, Formal analysis, Data curation. **Miracle Amadi:** Writing – review & editing, Visualization, Methodology, Formal analysis. **Baylor Fain:** Writing – review & editing, Visualization, Validation, Software, Methodology, Investigation, Formal analysis, Data curation. **Marcel Kouete:** Visualization, Investigation. **Rhys A. Farrer:** Writing – review & editing, Funding acquisition. **Amy J. Haeseler:** Writing – review & editing, Project administration. **Orly Razgour:** Writing – review & editing, Methodology, Funding acquisition. **Chris Richardson:** Writing – review & editing, Investigation. **Byron Weckworth:** Writing – review & editing, Investigation. **Julie Weckworth:** Funding acquisition. **Flora Whiting-Fawcett:** Writing – review & editing, Investigation. **Duncan Wilson:** Writing – review & editing, Funding acquisition. **Casey C. Day:** Writing – review & editing, Software, Funding acquisition, Data curation, Conceptualization.

## Consent for publication

Not applicable.

## Ethics approval

Not applicable.

## Funding

This research was supported by the 10.13039/100000001National Science Foundation (Grant No 2407848), 10.13039/501100000268Biotechnology and Biological Sciences Research Council UK (Grant No BB/Z517379/1), 10.13039/100000202US Fish and Wildlife Service (Grant No F23AP00383-00), 10.13039/100004930Texas Parks and Wildlife Department (Grant No F21AF03714), 10.13039/100000202US Fish and Wildlife Service (Grant No F25AP00448-00), National Institute of General Medical Sciences of the National Institutes of Health (P20GM130418), and computational resources from the 10.13039/100008524University of Montana's Computational Ecology Lab, Data and Modeling Core, and HellGate Shared Computing Cluster (Grant No 2018112 & 1925267). The funders of the study had no role in study design, data collection, data analysis, data interpretation, or writing.

## Declaration of competing interest

The authors declare that they have no known competing financial interests or personal relationships that could have appeared to influence the work reported in this paper.

## Data Availability

Source code at https://github.com/ComputationalEcologyLab/CDMetaPOP. All data supporting these findings are available at https://github.com/ComputationalEcologyLab/CDMetaPOP-Disease-Manuscript.
