# Supplementary material for: Simulating the landscape eco-evolution of host-pathogen systems with CDMetaPOP
Source: Ecol Inform. 2026 Aug;97:103892. doi: 10.1016/j.ecoinf.2026.103892 (PMC13469625; doi:10.1016/j.ecoinf.2026.103892)
Supplement: Supplementary file 1 [file mmc1.pdf]

Additional file for

Simulating the Landscape Eco-Evolution of Host-Pathogen Systems with CDMetaPOP

Authors

Erin L. Landguth, Allison Williams, Marissa Roseman, Miracle Amadi, Baylor Fain, Marcel Kouete, Rhys Farrer, Amy Haeseler, Orly Razgour, Chris Richardson, Byron Weckworth, Julie Weckworth, Flora Whiting-Fawcett, Duncan Wilson, Casey Day

Correspondence to: Erin Landguth erin.landguth@mso.umt.edu

This file includes:

Text A.1 - User manual description for implementing disease module

Table A.1 - List of parameters and options for implementing disease module

Figure A.1 - CDMetaPOP life cycle flow diagram.

Text A.1 - User manual description for how to model disease dynamics in CDMetaPOP (Section 5.4)
This section describes how to use the flexible, compartmental disease module within CDMetaPOP to simulate the spread of infectious diseases. To lower the "activation energy" for new users, we present this step-by-step workflow as a "Quick-Start" for implementing the CDMetaPOP-Disease module, moving from basic setup to complex eco-evolutionary scenarios.

Example Files: Example files can be found in the github repository with 'this study' (Landguth et al., in review) examples https://github.com/ComputationalEcologyLab/CDMetaPOP-Disease-Manuscript. We recommend navigating here and starting with ../Figures/Figure1/ and to follow along with the guide steps below.

Getting Started: A Basic SIR Model To begin modeling disease dynamics, you first need to activate and parameterize the module. This involves five main steps within the input files. If you would like to follow along with input files go to https://github.com/ComputationalEcologyLab/CDMetaPOP-Disease-Manuscript/Figures/Figure1/CDMetaPOP_inputs/SIR.

1. **Open the RunVars.csv file** This is the first master file to 'run' CDMetaPOP. Open RunVars.csv and ensure that this file points to the correct PopVars file in the 'Popvars' field.

File header in csv file	Description & Arguments	Example
Popvars	Points to the main population variables file.	popvars/PopVars_diseaseTests_SIR.csv

2. **Activate the Disease Module in PopVars.csv** Now navigate to your main population variables file (e.g., popvars/PopVars_diseaseTests_SIR.csv) that was specified in the RunVars file. Open the PopVars file and ensure 'implement_disease' field is not set to 'N'.

File header in csv file	Description & Arguments	Example
implement disease	Turns the disease module on and specifies when in the life cycle state transitions occur.<br><br>'N': Off (Default)<br>'Back': During the first DoUpdate() module<br>'Out': During the second DoUpdate() module<br>'Both': During both DoUpdate() modules	Back<br><br>"Disease state transitions occur during this time (e.g., back at natal grounds)."

3. **Specify the Disease Parameter File in PatchVars.csv** Next, for each patch (or set of patches), you must point to a file containing the specific disease parameters. This allows for spatial

variation in disease dynamics. CDMetaPOP requires the designated PopVars to reference a PatachVars files. Navigate to the PatchVars file (e.g., patchvars/PatchVars_diseaseTests_SIR.csv) and ensure the 'disease_file' field has the correct disease file.

File header in csv file	Description & Arguments	Example
disease_file	The path and name of the disease-specific parameter file for that patch.	otherfiles/disease/DiseaseVars_SIR.csv

4. **Define the Disease Dynamics in DiseaseVars.csv** This is the core file where you define the structure of your disease model. For a simple SIR model, you would specify three states and the initial proportion of the population in each. Open the DiseaseVars file example specified in the PatchVars file (above), e.g., otherfiles/disease/DiseaseVars_SIR.csv, check the following file headers and update information for your disease model.

File header in csv file	Description & Arguments	Example
Number of States	The total number of states. State 0 is always assumed to be the initial susceptible state.	3<br><br>"State 0 (*S*), State 1 (*I*), and State 2 (*R*)"
Initial Conditions	The proportion of individuals to be initialized in each state at t=0, separated by semicolons. Must sum to 1.	0.9;0.1;0.0<br><br>"90%, 10%, and 0% of the individuals within this patch are initialized as 'State 0', 'State 1', and 'State 2', respectively."
Transition Rates	The path and name of the transition matrix file.	/otherfiles/disease/TransitionMatrix_SIR.csv
Susceptible States	The state(s) considered susceptible.	0<br><br>"State 0 is considered the Susceptible State in this example."

Infection States	The state(s) considered infectious.	1<br><br>“State 1 is Infected State in this example.
Death States	The state(s) used for mortality.	N<br><br>“In this example, there are no individuals that move into a ‘death’ compartment and thus removed from the simulations.”
Initial Offspring States	How to initialize offspring states.<br>'Susceptible': All are born in state 0 (Default).<br>'Random': Drawn from initial conditions.<br>'Vertical_X:Y': Vertical transmission from infected mothers with mean rate X and standard deviation Y.	Susceptible<br><br>“All offspring are assigned State 0 in this example.”
Start Disease	The time step to initiated disease spread and ‘start’ the individual state transitions.	0
Transmission Mode	The specified transmission mode.<br><br>‘Direct’: Individual to individual transmission<br>‘Indirect’: Patch environmental contaminants to individual transmission. Note ‘Direct’ is also included here but can be removed or isolated out through the use of the Transition Matrix file.	Direct<br><br>“Only direct transmission is considered in this example, that is, host to host disease spread.”
Disease Resistant	The transition rate within the specified model to apply disease resistance that is linked to genotype ‘RR’.	N<br><br>“No disease defense strategy is considered in this example.”

Disease Tolerant	The transition rate within the specified model to apply disease tolerance that is linked to genotype 'TT'.	N<br><br>"No disease defense strategy is considered in this example."

5. **Create the Transition Matrix in TransitionMatrix_SIR.csv** Within the DiseaseVars file a transition matrix file is specified (e.g., otherfiles/disease/TransitionMatrix_SIR.csv). This file defines the mean transition rates of moving from one state to another. The matrix must be square (Number of states x Number of states). For a simple SIR model where individuals move from Susceptible (*S*, State 0) to Infected (*I*, State 1) at rate β, and from Infected (*I*, State 1) to Recovered (*R*, State 2) at rate γ, the file would look like this:

TO (rows) FROM (cols)

S	I	R	
0.0	0.0	0.0	
0.5	0.0	0.0	This is β, the S -> I transition rate
0.0	0.2	0.0	This is γ, the I -> R transition rate

Note: The S -> I transition rate (β) is treated as the transmission rate and is multiplied by the proportion of infected individuals (I/N) in the patch to determine the final probability of infection for a susceptible individual (i.e., frequency-dependent transmission). Check back soon for a density-dependent transmission option!

6. **Run the Basic SIR model:** If you followed the install instructions at the beginning of this user manual, ensure that your command prompt/Python interpreter of your choice is pointing to the directory for the CDMetaPOP source code. Then, run the example file by giving this 5-argument command:
   {RUN PYTHON} {Name or python file} {Directory location of example files} {Name of RunVars file} {Name of output folder to be created in the directory location of the example files}

   e.g.,

   python CDmetaPOP.py /Figures/Figure1/CDMetaPOP_inputs/SIR RunVars.csv Basic_SIR_Model

7. **See https://github.com/ComputationalEcologyLab/CDMetaPOP-Disease-Manuscript /Figures/Figure1/CDMetaPOP_inputs/ for additional example runs** for
   a. SEIR – this model adds an 'exposed' compartment and thus, 4 total compartments.
   b. SIRS – this model has 'recovered' individuals returning to 'susceptible' and 3 total compartments (note difference in TransitionMatrix.csv from SIR model).

c. SIRP – Technically a 4-compartment model, but only 3 compartments for host transitions and an additional compartment for an environmental reservoir. See next example.

Advanced Features: Indirect Transmission Models (Figure 1d from this study)

Beyond basic SIR models, the module allows for more complex scenarios, including transmission from the environment. For example, you can model indirect transmission - scenarios where individuals become infected from a pathogen in the environment (e.g., a contaminated water source or soil).

Example Files: Example files can be found in the github repository with 'this study' (Landguth et al., in review) https://github.com/ComputationalEcologyLab/CDMetaPOP-Disease-Manuscript/tree/main/Figures/Figure1/CDMetaPOP_inputs/SIRP.

Specifying Indirect Transmission in a SIRP model Follow all steps the same for setting up the SIR basic model from above. Additional changes are as follows:

1. **In DiseaseVars.csv**, set the 'Transmission mode' field to 'Indirect'. This enables the environmental reservoir functionality.

2. **In PatchVars.csv**, add a column Environmental Reservoir to set the initial pathogen load or contaminant value for each patch.

File header in PatchVars.csv	Description & Arguments	Example
Environmental Reservoir<br><br>"Env Res", "Pathogen Load", Aliases depending on the version of CDMetaPOP	The initial concentration/load of the pathogen in the patch's environmental reservoir. This column is only used when 'Indirect' transmission is specified in the DiseaseVars.csv file.	1.0

3. **In TransitionMatrix.csv**, add the last row/column information for transition rates for the environmental reservoir. For example, SIR-P model where individuals move from Susceptible (*S*, state 0) to Infected (*I*, state 1) at rate β_d for direct transmission, and from Infected (*I*, state 1) to Recovered (*R*, state 2) at rate γ. Then, individuals can shed to the environment by considering a transition rate from I, state 1, to Environmental Reservoir (*P*, state 3) at rate α and vital dynamics on the P, state 3, can be considered through μ. The file would look like this:

TO (rows) FROM (cols)

S	I	R	P	
0.0	0.0	0.0	0.0	
0.5	0.0	0.0	0.9	This is β, the S -> I transition rate for direct and indirect (P -> I)

0.0	0.2	0.0	0.0	This is γ, the I -> R transition rate
0.0	0.0	0.0	-0.1	This is μ, the decay rate for P

<u>Advanced Features</u>: Modeling Genetics of Disease Response (Resistance & Tolerance) (Figure 4 from this study)

Beyond basic SIR models, the module allows for more complex scenarios, including the evolution of host disease responses. A key feature of this module is the ability to link an individual's genotype to disease parameters, allowing you to model the evolution of disease response strategies like resistance (reducing infection probability) or tolerance (reducing negative fitness effects).

Example Files: https://github.com/ComputationalEcologyLab/CDMetaPOP-Disease-Manuscript/tree/main/Figures/Figure4/CDMetaPOP_inputs

By setting up these parameters, you can simulate how selection acts on these loci, potentially leading to the spread of resistance or tolerance alleles through the population and altering the course of the epidemic.

1. **In DiseaseVars.csv**, you must first specify which transition rate in your model corresponds to a disease response strategy. Use the Transition rate between states parameter. For example, to model resistance that reduces the S -> I transition, you would set this parameter to 0_1.

File header in DiseaseVars.csv	Description & Arguments	Example
Transition rate... #1	Specifies which transition (FROM_TO) is governed by the first disease response locus.	0_1
Transition rate... #2	Specifies which transition (FROM_TO) is governed by the second disease response locus.	1_3

2. **In PatchVars.csv**, you then provide the specific transition rates for each genotype at that locus. This requires three columns for each disease response locus, corresponding to the three possible genotypes (e.g., AA, AB, BB). The values in these columns will override the single value provided in the TransitionMatrix.csv file for the specified transition.

File headers in PatchVars.csv	Description & Arguments	Example
dd1_AA, dd1_AB, dd1_BB	The transition rates for individuals with each genotype at the first disease	0.1, 0.4, 0.8

	response locus. The column headers must be unique.	
dd2_AA, dd2_AB, dd2_BB	The transition rates for individuals with each genotype at the second disease response locus.	0.05, 0.05, 0.2

<u>Analyzing Outputs</u>: The disease module generates two primary types of output files to allow for multi-scale analysis of epidemic dynamics:

1. Individual-level Output (ind{year}.csv): A new column titled state is added to the standard individual output files. This records the integer disease state (0, 1, ..., *i*) for every individual in the simulation at that specific time step. State 0 is always the initial susceptible state.

2. Population-level Summary (summary_popAllTime_DiseaseStates.csv): This dedicated summary file tracks aggregate counts of individuals in each state across the entire simulation area (or per patch) at various stages of the life cycle.

Output Column	Description
**Year**	The simulation time step.
**N_Initial**	Total individuals at the start of the simulation time step.
**States_GetMetrics**	Counts of individuals in each state (S_0; S_1; ...; S_i) at the start of the time step.
**States_AfterAnyAddedInds**	Count of individuals in each state after individuals are added to the simulations.
**States_SecondUpdate**	Counts of individuals in each state after the demographic and epidemiological transition module has run (i.e., second DoUpdate()).
**States_SecondUpdate_AfterDeathsRemoved**	Final state counts after disease-induced and demographic mortality have been processed.
**N_AfterEmigration**	Total individuals after the Emigration() module.
**States_AddedAge0s**	Counts of newborns (Age 0) assigned to each state (useful for tracking vertical transmission).
**N_AfterEmiMort**	Total individuals after the density independent mortality module.
**States_ThirdUpdate**	Counts of individuals in each state after the demographic and epidemiological transition module has run (i.e., third DoUpdate()).
**States_ThirdUpdate_AfterDeathsRemoved**	Final state counts after disease-induced and demographic mortality have been processed.

**N_afterImmigration**	Total individuals after the Immigration() module.
**N_AfterImmiMort**	Total individuals after the density independent mortality module.
**Ct**	Effective transmission rate (Ct)<br><br>Ct = (count of new infected during timestep) / (current timestep count of infected * current timestep number of Susceptible) * current total population size

Note: ‘|’ separates patches, with the first index the sum of all patches: TOTAL | Patch 1 | Patch 2 | …
Note: Order of columns follows module progression of CDMetaPOP time loop

Table A.1. List of all input parameters in each of the 4 primary input files along with example inputs and explanations for each option. ✝Indicates a parameter that can vary over time using pipe delimiter (‘|’). Please note that Python counts from 0, so that the first state would be indicated with ‘0’, the second state would be indicated by ‘1’, and so on.

**File header in csv file**	**Input csv file**	**Description with possible arguments**
Implement_disease	PopVars	The answer to turn disease processes on and where in CDMetaPOP’s life cycle they are applied.<br><br>‘N’ - Do not implement disease module<br>‘Back’ - Implement disease module in first DoUpdate()<br>‘Out’ - Implement disease module in second DoUpdate()<br>‘Both’ - Implement disease modules in both DoUpdate()
Disease_file	PatchVars	The location and file name for each patch of the disease-specific parameters and options. Note, each patch can link to a different or same DiseaseVars file.<br><br>‘../diseasefiles/DiseaseVars.csv’ - Example of named csv file ‘DiseaseVars.csv’ located in a folder ‘diseasefiles’.
Environmental Reservoir<br><br>Our aliases depending on the version of CDMetaPOP	PatchVars	When indirect transmission is specified, then this value is used for the population of the environment reservoir (containment value, pathogen load, etc). This column is ignored unless ‘indirect’ is specified in the DiseaseVars file.<br><br>‘1’ - Any float value is accepted here.
Disease Response #1 genotype<br><br>“’CC’, ‘Cc’, ‘cc’, ‘Resistant_CC’, ‘Resistant_Cc’, ‘Resistant_cc’, Aliases Depending on the version of CDMetatPOP”	PatchVars	3 additional columns are used to specify what transition value should be used when this disease response mechanism is specified (in the DiseaseVars file). For example, CC - 0.8,<br>Cc - 0.1, cc - 0.1 would say that individuals with the given allele combination at that locus would have the given transition rate.
Disease Response #2 genotype	PatchVars	3 additional columns are used to specify what transition value should be used when this disease response

“’’DD, ‘Dd’, ‘dd’, ‘Resistant_DD’, ‘Resistant_Dd’, ‘Resistant_dd’, Aliases Depending on the version of CDMetatPOP”		mechanism is specified (in the DiseaseVars file). For example, DD - 0.8,<br>Dd - 0.8, dd - 0.1 would say that individuals with the given allele combination at that locus would have the given transition rate.
Number of states	DiseaseVars	The total number of states considered.<br><br>‘3’ - Consider 3 states for 0, 1, and 2. 0 will always be considered as the Susceptible state.
Initial Conditions	DiseaseVars	The distribution used to initialize an individual's state at t = 0. A binomial outcome is simulated for each individual.<br><br>‘0.9;0.1;0.0’ - 90% of the population will be in state 0, 10% of the population in state 1, and 0% of the population in state 2. Note, this distribution must sum to 1.
Transition Rates	DiseaseVars	The location and file name for the transition matrix. This file must be square of size ‘number of states’ x ‘number of states’. Rows read TO and columns read FROM.<br><br>‘../diseasefiles/TransitionMatrix_SIR.csv’ - Example of named csv file for ‘TransitionMatrix.csv location in the folder ‘diseasefiles’.
Infection states	DiseaseVars	Indicate what state(s) are involved in the infection process.<br><br>‘1’ - State 1 indicates the infected individuals, e.g., SIR.<br>‘2’ - State 2 indicates the infected individuals, e.g., SEIR.<br>Note, State 0 will be assumed to be the susceptible individuals, S.
Death states	DiseaseVars	Indicate what state(s) are involved in death. That is, if this state compartment is given, these individuals will be removed from the population, and the population will no longer be in equilibrium (N does not have to equal 0).<br><br>Default = 1

		‘3’ - State 2 indicates the individuals that will be removed from the simulation, e.g., SIRD.
Initial Conditions Offspring	DiseaseVars	Indicate what option to use for initializing the offspring (age 0) states.<br><br>Default = ‘Susceptible’<br><br>‘Susceptible’ - All offspring added to the population will be assigned state 0.<br>‘Random’ - Offspring will be initialized from the ‘State initialize conditions’ field given in this file.<br>‘Vertical_0.1:0.05’ - Vertical transmission with mean rate 0.1 and standard deviation 0.05. Infected state will be used to initialize the offspring’s state if a) the mother was infected and a random draw outcome is less than 0.1. When the standard deviation is specified, then at the beginning of each time step, this variable will be updated around the given mean and standard deviation.
Start Disease	DiseaseVars	Indicate the time step at which the disease module will be turned on.<br><br>Default = 0<br><br>‘0’ - Initialize disease module upon start of simulations.<br>‘10’ - Initialize disease module after some amount of burn-in time 10.
Transmission mode	DiseaseVars	Transmission mode for modeling. Note that direct transmission will always be assumed. If only indirect transmission is wished, then direct transmission rates can be set to 0 in the transition matrix.<br><br>Default = ‘Direct’<br><br>‘Direct’ - Direct transmission from host to host.<br>‘Indirect’ - Indirect transmission from environment to host/individual.

Disease Resistant<br><br>"Transition rate between states X_Y that correspond to disease response strategy #1"	DiseaseVars	Indicate the disease transition rate that this disease response strategy operates on. Note, if this option is used, then the PatchVars values for the transition rate will be used for each individual with the given genotype (i.e., the Transition Matrix file will ignore the given transition rate, but instead use the values in the PatchVars).<br><br>Default = 'N'<br><br>'N' - No disease response is on and associated with genotypes<br>'X_Y' - e.g. 0_1 would correspond to the transition rate FROM state 0 TO state 1 given in the transmission matrix file that would be modified with given genotype. Genotype - disease transition rate values are then given in the PatchVars file.
Disease Tolerant<br><br>"Transition rate between states that correspond to disease response strategy #2"	DiseaseVars	Indicate the disease transition rate that this disease response strategy operates on.<br><br>Default = 'N'<br><br>'N' - No disease response is on and associated with genotypes<br>'X_Y' - e.g. 0_1 would correspond to the transition rate FROM state 0 TO state 1 given in the transmission matrix file that would be modified with given genotype. Genotype - disease transition rate values are then given in the PatchVars file.

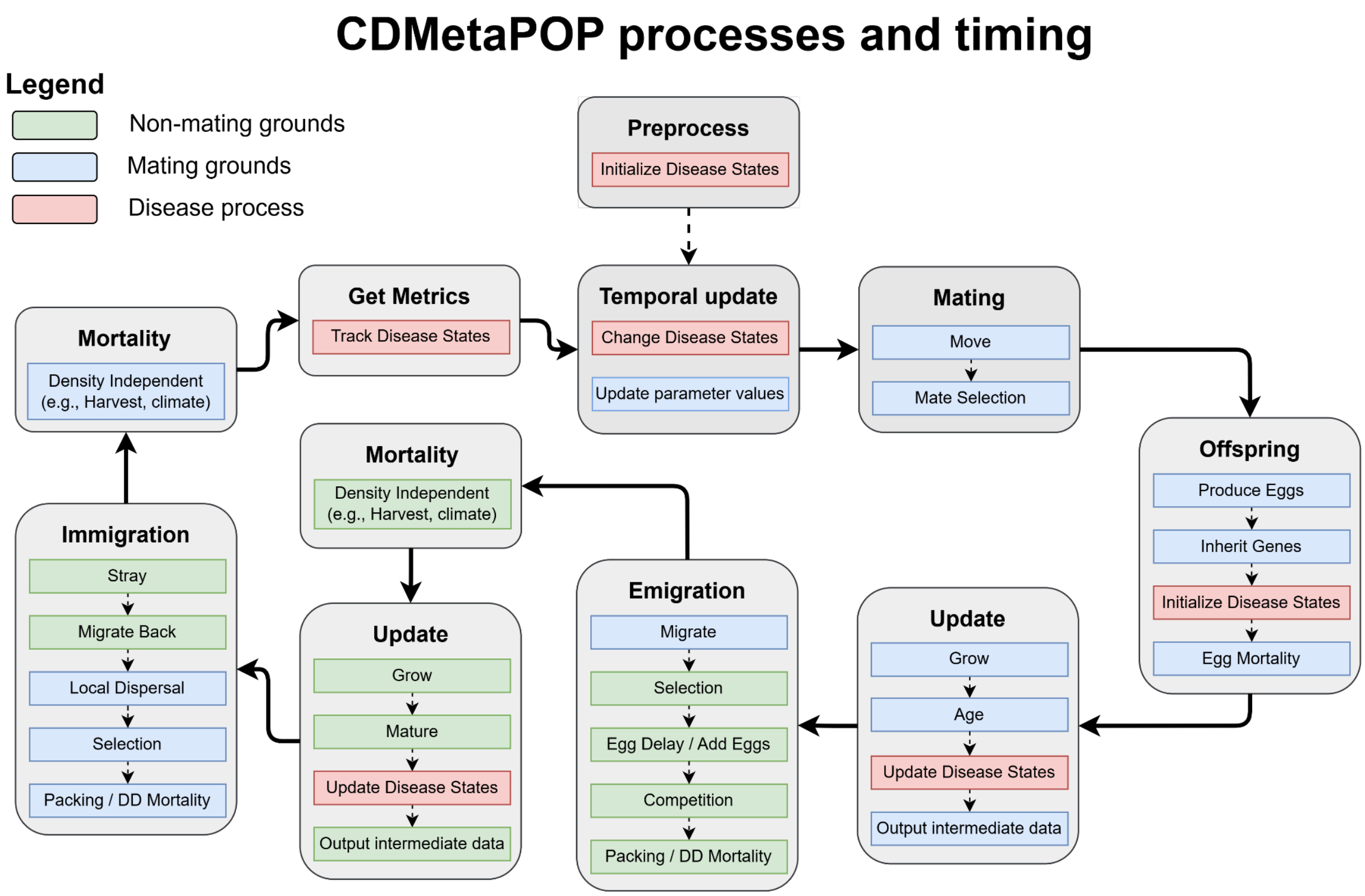

Figure A.1 - CDMetaPOP's basic process cycle and flow diagram. Rectangle boxes illustrate CDMetaPOP existing modules and parallelograms highlight specific functions. Red parallelograms point towards new disease functions. Initialization of disease states occurs at the model onset (PreProcess()) and also when offspring join the population (DoOffspring()). Disease states are updated in either one or both of the DoUpdate() modules. Disease variables can be modified through time (CDClimate()) and tracking of disease states occurs throughout the program, e.g., GetMetrics(). Green and blue shapes point towards different life history strategies and split the time unit between two different sub-units. See user manual for more details.
